# Detection of Transcranial Alternating Current Stimulation Aftereffects Is Improved by Considering the Individual Electric Field Strength and Self-Rated Sleepiness

**DOI:** 10.3389/fnins.2022.870758

**Published:** 2022-06-27

**Authors:** Iris Steinmann, Kathleen A. Williams, Melanie Wilke, Andrea Antal

**Affiliations:** ^1^Department of Cognitive Neurology, University Medical Center Göttingen, Göttingen, Germany; ^2^Max Planck Institute for Human Cognitive and Brain Sciences, Leipzig, Germany; ^3^German Primate Center, Leibniz Institute for Primate Research, Göttingen, Germany; ^4^Department of Neurology, University Medical Center Göttingen, Göttingen, Germany

**Keywords:** tACS (transcranial alternating current stimulation), alpha amplitude, subject-specific variability, electrical field strength, sleepiness, aftereffect

## Abstract

Non-invasive electrical stimulation methods, such as transcranial alternating current stimulation (tACS), are increasingly used in human neuroscience research and offer potential new avenues to treat neurological and psychiatric disorders. However, their often variable effects have also raised concerns in the scientific and clinical communities. This study aims to investigate the influence of subject-specific factors on the alpha tACS-induced aftereffect on the alpha amplitude (measured with electroencephalography, EEG) as well as on the connectivity strength between nodes of the default mode network (DMN) [measured with functional magnetic resonance imaging (fMRI)]. As subject-specific factors we considered the individual electrical field (EFIELD) strength at target regions in the brain, the frequency mismatch between applied stimulation and individual alpha frequency (IAF) and as a covariate, subject’s changes in mental state, i.e., sleepiness. Eighteen subjects participated in a tACS and a sham session conducted on different days. Each session consisted of three runs (pre/stimulation/). tACS was applied during the second run at each subject’s individual alpha frequency (IAF), applying 1 mA peak-to-peak intensity for 7 min, using an occipital bihemispheric montage. In every run, subjects watched a video designed to increase in-scanner compliance. To investigate the aftereffect of tACS on EEG alpha amplitude and on DMN connectivity strength, EEG data were recorded simultaneously with fMRI data. Self-rated sleepiness was documented using a questionnaire. Conventional statistics (ANOVA) did not show a significant aftereffect of tACS on the alpha amplitude compared to sham stimulation. Including individual EFIELD strengths and self-rated sleepiness scores in a multiple linear regression model, significant tACS-induced aftereffects were observed. However, the subject-wise mismatch between tACS frequency and IAF had no contribution to our model. Neither standard nor extended statistical methods confirmed a tACS-induced aftereffect on DMN functional connectivity. Our results show that it is possible and necessary to disentangle alpha amplitude changes due to intrinsic mechanisms and to external manipulation using tACS on the alpha amplitude that might otherwise be overlooked. Our results suggest that EFIELD is really the most significant factor that explains the alpha amplitude modulation during a tACS session. This knowledge helps to understand the variability of the tACS-induced aftereffects.

## Introduction

Cognitive and behavioral processes go along with highly coordinated spatiotemporal activity patterns within and between neurons and neuronal networks. Most of these actions should be synchronized by coherent membrane potential oscillations in order to reach optimal large-scale systemic functions. Neural oscillations are involved in many brain functions and are thought to be responsible for the ongoing neural communication, and also contributes to synaptic plasticity, a perceptual neural process underlying learning and long-term memory functions (for a review see: Fries, 2005; Watson and Buzsáki, 2015; Singer, 2018; Womelsdorf and Hoffman, 2018). In electroencephalography (EEG) measures of human brain activity, alpha oscillations (8–12 Hz) constitute the most dominant brain rhythm in the awake state, and are thought to be involved in a multitude of cognitive functions such as memory, attention and perception (Foxe and Snyder, 2011; Klimesch, 2012). Alpha oscillations play a significant role in the integration and regulation of brain network activities (Sadaghiani et al., 2010; Helfrich et al., 2017; Clayton et al., 2018b). The correlative link between alpha oscillations and various cognitive aspects has been successfully established using non-invasive electro- and magnetoencephalography (EEG/MEG) (Klimesch, 1999; Klimesch et al., 2007; Van Diepen et al., 2019) measures, as well as with intracranial recordings in epileptic patients (Haegens et al., 2015; Chapeton et al., 2019). Another strategy to assess the potential roles of these oscillations in causing the cognitive changes is to manipulate them and to measure the effect of the modulation on neuronal activity and behavior.

In the past decade, transcranial alternating current stimulation (tACS) gained increasing attention as a non-invasive brain stimulation technique to manipulate neuronal oscillations in a frequency-specific manner, by externally applying sinusoidal currents to the scalp (Antal et al., 2008; Antal and Herrmann, 2016). A relatively long lasting aftereffect, that persists when stimulation has ended, is of specific interest for research, particularly for clinical interventions (Ahn et al., 2019; Elyamany et al., 2021). The potential of tACS to modulate neuronal oscillations has already been shown in animal models, using invasive measurements (Fröhlich and McCormick, 2010; Opitz et al., 2016). In humans, assessment of tACS effects has been limited to behavioral measures, such as performance measurements and neurophysiological aftereffects, including EEG and functional magnetic resonance imaging (fMRI), with the studies very frequently yielding inconsistent results. For example, several studies showed persistent aftereffects on occipital alpha power (measured by EEG) lasting for 30–70 min after tACS targeting the visual cortex (Zaehle et al., 2010; Neuling et al., 2013; Kasten et al., 2016; Haberbosch et al., 2019), while a number of other studies did not find such aftereffects (Clayton et al., 2018a; Fekete et al., 2018; Stecher and Herrmann, 2018). Indeed, the efficiency of tACS to modulate behavior via manipulating the amplitude of brain oscillations varies strongly between subjects and studies (Thut et al., 2017; Veniero et al., 2017). This inconsistency reprepresents a substantial limitation for tACS in research and potential clinical applications (Wu et al., 2021). Furthermore, although findings from several studies suggest that tACS has an effect on brain oscillations and related behavior, there is also an increasing skepticism based on an absence of reproducible results across research groups, and frequent reports of null effects at the behavioral level and also on neuronal activity (Veniero et al., 2017; Wittenberg et al., 2019). Therefore, doubts have been raised in the brain stimulation community regarding the effectiveness of low intensity tACS (Lafon et al., 2017; Ergo et al., 2020), and it has been suggested that much higher intensities than have been used in the past are needed to modulate ongoing neuronal activity (Vöröslakos et al., 2018).

There are several subject-specific factors, for example anatomical and physiological factors, age, gender, brain state, hormonal levels, and pre-existing regional excitability, which could confound the effects of tACS (see e.g., Feurra et al., 2013; Neuling et al., 2013; Krause and Cohen Kadosh, 2014; Benwell et al., 2015; Alagapan et al., 2016; Kasten and Herrmann, 2020). These factors can at least partly explain the variable effectiveness of tACS between subjects within a study, but also the various outcomes between studies. In addition, more efforts have recently been made to evaluate the impact of individual methodological factors on tACS aftereffects (Stecher and Herrmann, 2018; Kasten et al., 2019). Kasten et al. (2019), for example, reported the benefit of considering subject-specific factors, such as electrical field (EFIELD) strength, precision of targeting a given region and the precision of the stimulation frequency. Considering these individual factors in a multiple linear regression model, they were able to explain a large amount of variability in tACS aftereffects among subjects (Kasten et al., 2019).

In the current study we aimed to investigate inter-subject variability of tACS aftereffects on the EEG alpha amplitude as well as on the within-network connectivity strength of the default mode network (DMN). The DMN is defined as a group of brain areas that are coherently active during rest. The connectivity strength of the network nodes is determined as the correlation of the blood oxygen level dependent (BOLD) signal recorded in fMRI. The network strength of the DMN is strongly associated with different types of internally directed mental processes, for example mind wandering and autobiographical memory (Gusnard et al., 2001; Fox et al., 2018; Buckner and DiNicola, 2019). These mental states are in turn coupled to modulations in the alpha frequency band (Braboszcz and Delorme, 2011; Compton et al., 2019). Indeed, a positive correlation of DMN activity and posterior alpha band activity has been shown in several combined EEG/fMRI studies (Scheeringa et al., 2012; Mo et al., 2013). Specifically, the two medial network nodes, the posterior cingulate cortex (PCC) and the medial prefrontal cortex (mPFC) of the DMN, were found to be associated with the modulation of the oscillatory activity in the alpha range that correlated with sleepiness (Pomares et al., 2019). In a recent study, Clancy et al. (2022) observed in their fMRI data a significant connectivity change between these network regions after application of alpha-tACS. Administering tACS at the individual peak alpha frequency between 8 and 12 Hz over four consecutive days, they found immediate and lasting (more than 1 day) increases in resting-state posterior – frontal connectivities in the alpha frequency range.

The results of previous studies suggest that tACS can engage endogenous rhythms differently, depending on the pre-existing power of the targeted oscillation (Neuling et al., 2013; Alagapan et al., 2016). Indeed, the impact of the “state of the brain” before and during stimulation is a frequently discussed issue with regard to the variability of aftereffects induced by different kinds of brain stimulation methods (Antal et al., 2008; Silvanto et al., 2008; Bergmann, 2018; Feurra et al., 2019). However, aside from the fact that mental state can influence the effect of tACS, it could also be that a change in the mental state associated with the frequency band to be modified, masks the effect of the stimulation.

Our hypothesis was that the individual differences in the aftereffects refer to various tACS parameters, such as subject-specific EFIELD strength, or mismatch between IAF and stimulation frequency. With regard to sleepiness, we aimed to investigate the role of sleepiness only as a covariate that influences the alpha amplitude and DMN connectivity strength during the experiment in addition to tACS, asking the question, whether and how sleepiness-induced changes can cover (or mask) the effect of tACS. We recorded EEG and fMRI data simultaneously at different time windows [*pre* (before stimulation), *stimulation* (during stimulation), and *post* (after stimulation)] relative to the tACS or sham stimulation in two sessions on different days. During the experiment, the subjects watched a video designed to encourage in-scanner compliance for fMRI experiments (Vanderwal et al., 2015). We expected a stronger increase of subject’s occipital alpha EEG amplitude as well as in mPFC-PCC connectivity strength from *pre* to *post* recording during the tACS session in comparison to sham stimulation. Further, we aimed to model inter-subject variability of *pre* to *post* changes of both outcome variables (alpha amplitude and mPFC-PCC connectivity strength) integrating subject-specific parameters, such as tACS EFIELD strength, frequency mismatch between stimulation frequency and IAF and self-rated sleepiness, in a multiple linear regression model.

## Materials and Methods

### Participants

Twenty-two subjects (13 females, mean age 24.2 years) gave written informed consent before participating. All subjects were university students and received payment for participation. They were healthy, medication-free on the day of recording, and reported no presence of neurological or psychiatric disorders. Each subject participated in two blinded experimental sessions, a tACS and a sham session conducted at least 1 week apart, in randomized order between subjects. To control for time-of-day influence of effects (Wong et al., 2018), experiments were restricted to the morning hours. The study was approved by the local Ethics Committee of the University Medical Center Göttingen and performed according to the Declaration of Helsinki.

### Study Protocol

**FIGURE 1:**
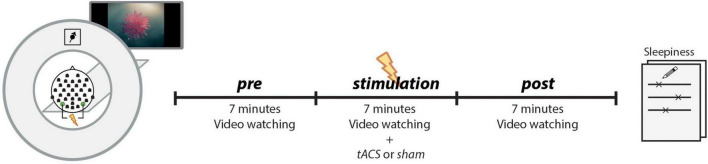
EEG and fMRI data were simultaneously recorded during three runs (*pre*, *stimulation*, *post*), while subjects passively watched the same video each run. During the second run (stimulation), subjects received a continuous stimulation at their IAF (tACS-session) or only a 10-s ramp-up/ramp-down stimulation (sham-session). The tACS electrodes were placed at PO7/PO8 positions, and the EEG was recorded from 31 electrodes. After the experiment, subjects retrospectively rated their sleepiness during each run on a continuous line ranging from *sleepy* to *very awake*.

The experimental procedure is depicted in Figure 1. Before the subject entered the MRI scanner for the main experiment, a 3-min resting-state EEG was recorded, with the participant seated with eyes closed. These data were used to determine the subject’s IAF (see section “*Individual alpha frequency”*), which was used as the stimulation frequency. The main experiment took place in the MRI scanner and followed the same procedure in both sessions (tACS and sham). EEG and fMRI were recorded simultaneously, while the subjects watched a 7-min video without sound that was repeated for each of the three runs (*pre*, *stimulation*, *post*). The video consisted of abstract forms reshaping and moving without narrative or cuts and was developed to keep participants alert and to generate common brain states across subjects during resting state fMRI (Vanderwal et al., 2015). The subjects received stimulation only during the second run (7 min in the tACS session, 10 s during sham session). After the experiment, the subjects completed a questionnaire to assess sleepiness and stimulation sensation during each run.

### Data Acquisition

#### Electroencephalography

Electroencephalography data were recorded with a MR-compatible EEG cap with 31 sintered Ag/AgCl electrodes mounted on an elastic cap (10–20 system) (Easycap, Falk Minow, Munich, Germany). An additional electrode was attached on the subject’s back to record the electrocardiogram (ECG). The electrode impedance was kept below 20 kΩ. The signal was recorded using BrainVision Recorder software (Brain Products GmbH, Gilching, Germany) with an online bandpass filter (0.016–250 Hz). EEG data were sampled at a rate of 5,000 Hz and amplified using a BrainAmp MR plus amplifier (Brain Products, Munich, Germany).

#### Functional Magnetic Resonance Imaging

All images were acquired using a 3 Tesla Magnetom Prisma scanner (Siemens Healthcare, Erlangen, Germany) with a 64-channel phased-array head coil. In the first 7–8 min, adjustment scans and a high-resolution T1-weighted anatomical scan (three-dimensional (3D) turbo fast low angle shot; repetition time (TR): 2,250 ms, inversion time (TI): 900 ms, echo time (TE): 3.3 ms, flip angle 9°, isotropic resolution of 1 mm × 1 mm × 1 mm) were recorded. During the further procedure, functional data were acquired using gradient-echo, echo-planar imaging with T2*-weighting (TR: 2 s, TE: 30 ms, flip angle 50°, 35 slices of 3-mm thickness, gap between slices of 20%, in-plane resolution of 3 mm × 3 mm). A total of 210 whole-brain volumes were acquired in each functional run. Before every functional scan, an additional scan with the same parameters but opposite phase encoding direction was recorded to correct for distortion in the functional data.

To improve the individualized volume conduction model for electrical field simulation (see section “*electrical field simulation”*), we recorded additional anatomical scans in a separate session, including a T2-weighted anatomical scan (3D turbo spin echo; TR: 3500 ms, TE: 282 ms, variable flip angle, with integrated parallel acquisition technique: factor 2, isotropic resolution of 1 mm × 1 mm × 1 mm, no fat saturation), and diffusion-weighted MRI performed using a spin-echo echo planar imaging technique at 1.7 mm isotropic resolution (repetition time = 10,000 ms, echo time = 88 ms, parallel acquisition factor 2; acquisition matrix: 128 × 128, 75 slices), acquiring 64 image volumes with diffusion weighting (along 64 diffusion directions, *b* = 1,000 s/mm^2^) and one reference image without diffusion weighting.

Due to technical problems, T2 images are lacking in seven subject datasets. The T2 contrast was used to improve brain matter segmentation [skin, skull, cerebral spinal fluid (CSF), white matter, gray matter] for calculating the volume conduction model needed for tACS electrical field simulation.

#### Questionnaire

After each experiment, the subject completed a questionnaire designed to assess their sleepiness during the experiment and get information of possible sensation effects of the stimulation. Sleepiness was rated for each run with a handwritten mark on a 10-cm long line representing a continuum between *very awake* and *very sleepy* [Visual analogue scale (VAS); Funke and Reips, 2012].

### Transcranial Alternating Current Stimulation Application

A battery-driven NeuroConn DC-stimulator Plus (NeuroConn GmbH, Ilmenau, Germany) was used to deliver tACS through a pair of conductive rubber electrodes attached with electrode paste (Weaver and Company, Aurora, CO, United States) on the subject’s scalp. To target the occipital cortex, two round electrodes (2 cm diameter) were placed on the PO7 and PO8 positions, using the international 10–20 system. For a similar bilateral occipital montage, previous studies showed an increasing effect on the alpha amplitude (Zaehle et al., 2010; Vossen et al., 2015). All subjects received a sinusoidal stimulation of 1 mA (peak-to-peak) at their IAF, while impedance was kept below 20 kΩ. tACS was applied with 16 bits frequency resolution: The IAF was determined from the 3-min eyes-closed resting-state EEG recording before the main experiment. Stimulation was applied during the second run and lasted 7 min in the active tACS session, while the sham session consisted of just 10 s of stimulation at the beginning of the second run, to give an illusion of being stimulated (Kasten et al., 2019). Stimulation was always ramped up to maximum in 10 cycles at the beginning and ramped down in ten cycles at the end.

### Data Analysis

#### Software

The data were analyzed using MATLAB R2015b (The MathWorks Inc, Natick, MA, United States). For analyzing the EEG data, we used the Fieldtrip toolbox (Oostenveld et al., 2010) and EEGLAB (Delorme and Makeig, 2004) in combination with the FMRIB plug-in provided by the University of Oxford Centre for Functional MRI of the Brain (FMRIB) (Niazy et al., 2005). FMRI data were analyzed using FSL (Smith et al., 2004; Jenkinson et al., 2012) for top-up correction, and the CONN toolbox (Whitfield-Gabrieli and Nieto-Castanon, 2012) for further analysis steps. For electrical field stimulation we used SimNibs (Windhoff et al., 2013; Thielscher et al., 2015).

#### Electroencephalography Preprocessing

Gradient and cardioballistic artifacts were attenuated using optimal basis sets (OBS) as implemented in FMRIB Plug-in for EEGLAB (Delorme and Makeig, 2004; Niazy et al., 2005). Further analysis steps were performed using the Fieldtrip toolbox (Oostenveld et al., 2010). Data were downsampled to 1,000 Hz and low-pass filtered at 160 Hz. An independent component analysis (ICA) (*runica* method, 20 components) was calculated on a band-pass filtered version (1–20 Hz) of concatenated *pre* and *post* EEG data (separately per session). Components representing typical spatial topographies of blinks, saccades, residuals of cardioballistic artifacts or residuals of gradient-related artifacts were visually identified (on average three to five components per subject). The mixing matrix of the ICA decomposition model was then applied to the 160 Hz low-pass filtered data. Subsequently, the artifactual components were removed before the data were back-projected to channel space. These data were then re-referenced to an average of all channels and visually inspected, whereas artifact-contaminated parts were marked for later removal.

#### Individual Alpha Frequency

The stimulation frequency was determined from the 3-min eyes-closed recording period before the main experiment started (see section “*study protocol”*). Using BrainVision Analyzer 2.0 (Brain Products GmbH, Gilching, Germany), the EEG data were fast Fourier transformed with a frequency resolution of 0.2 Hz, and the frequency with the most dominant peak between 8 and 12 Hz from the occipital electrodes (O1, Oz, O2, POz) was visually identified. This frequency was used as the stimulation frequency. Since the IAF peak location can vary slightly across conditions, e.g., for eyes-closed and watching the video and also from day to day, we calculated an averaged IAF from all EEG data recorded during the video for further data analysis. Therefore, artifact-free EEG segments of *pre* and *post* in both sessions were split into 10 s epochs. Next, a Fourier transformation was calculated [0.1 Hz resolution, 0.5 Hz smoothing, multi taper (dpss)] for the Oz and POz electrodes and subsequently averaged over both channels. The frequency with the highest peak between 8 and 12 Hz was taken as IAF.

#### Alpha Amplitude

To evaluate changes of alpha activity between *pre* and *post* and between sessions, we determined the alpha amplitude as follows: a Hilbert transformation of preprocessed EEG data (bandpass filtered ± 2 Hz around IAF, 4th order Butterworth filter with *hamming* window) was calculated for Oz and POz channels separately. The absolute values of the complex Hilbert transformation, which represented the envelope of the filtered signal, were averaged in artifact-free segments over time and over both channels (Nelli et al., 2017). For each subject we calculated the difference in the alpha amplitude change between the two sessions (ΔΔ ALPHA_AMP) as Δ alpha amplitude (*post* - *pre*) calculated for the tACS session minus Δ alpha amplitude (*post* - *pre*) calculated for the sham session.

#### Functional Magnetic Resonance Imaging Preprocessing

In a first step, fMRI data were top-up (Andersson et al., 2003) corrected using FSL (Smith et al., 2004; Jenkinson et al., 2012) and further analyzed using the CONN toolbox (Whitfield-Gabrieli and Nieto-Castanon, 2012). After correction for motion, slice timing correction and outlier detection, functional data were spatially normalized into MNI space. The data were then downsampled to 2 × 2 × 2 mm and smoothed with a Gaussian kernel (6 mm FWHM). In a subsequent denoising step, six motion regressors, the mean signals of white matter and CSF, and a various number of noise components (one for each detected outlier) were regressed out of the data. Finally, functional data were band-pass filtered between 0.008 and 0.24 Hz.

#### Relation of Alpha Amplitude and Blood Oxygen Level Dependent Signal

To determine the voxelwise cortical regions that are related to the EEG alpha band activity recorded on the scalp, we performed an EEG-guided fMRI analysis. A general linear model (GLM) was calculated for every voxel of the preprocessed fMRI data, using a regressor derived from the EEG alpha amplitude. For each run of each subject, an alpha-related regressor was calculated using the signals from the Oz and POz electrodes. Separately, the continuous (7 min) EEG signals were Hilbert-transformed (bandpass filtered ± 2 Hz centered around IAF, 4th order Butterworth filter with *hamming* window) and their absolute values were convolved with the hemodynamic response function (HRF). Subsequently, the time courses were downsampled according to the fMRI acquisition rate (0.5 Hz) and, finally, averaged between electrodes.

Results of the EEG-guided fMRI analysis were then used to create binary masks to determine the electrical field strength at alpha related occipital voxels (see section “*electrical field simulation”*). For each subject, we created a binary mask that included the 1,000 occipital gray matter voxels showing the strongest (negative) alpha-BOLD correlations (MASK_alphaBOLD_). We focused on only negative correlations because previous studies have shown that in occipital areas, BOLD signal and occipital alpha amplitude are inversely related (Goldman et al., 2002; Laufs et al., 2003, 2006). Then, the first-level results of all runs (in total four: *pre* and *post* of tACS sessions plus *pre* and *post of sham session*s) were averaged separately per subject. The voxels in the occipital-parietal areas with the most negative *t*-values were determined individually for every subject and served as a binary mask for further analysis steps.

#### Default Mode Network Connectivity

To summarize DMN connectivity strength, mPFC-PCC functional connectivity was calculated for each run of each subject using a ROI-based correlation approach. ROIs were defined as 10 mm spheres centered around MNI coordinates adopted from CONN’s ICA analyses of the HCP dataset (497 subjects) [mPFC (1,55,–3) and the PCC (1,–61,38)]. For each ROI, the BOLD time course was averaged over all voxels. Connectivity was calculated as the *Spearman* correlation between the two ROI time courses and subsequently Fisher-z-transformed (Chang et al., 2013; Clancy et al., 2022). Changes in network connectivity within a session were calculated as the difference between the Fisher-z-transformed correlation factor from that session’s *post* and *pre* (yielding Δ CONNECTIVITY_tACS_ and Δ CONNECTIVITY_sham_). The difference in DMN connectivity strength between conditions (ΔΔ CONNECTIVITY) was calculated as Δ CONNECTIVITY_tACS_ minus Δ CONNECTIVITY_sham_.

#### Self-Rated Sleepiness

For each run, subjects reported their experienced degree of sleepiness by marking the appropriate point on a continuous line with extremes representing *very sleepy* and *very awake*. The marked position was quantified in relation to the full length of the line giving a sleepiness value between 0 and 1 for every run. In the first step we determined the change of sleepiness during each session by subtracting the *pre* sleepiness value from the *post* sleepiness value. In the next step these results were subtracted from each other to determine the difference in sleepiness change between the tACS and the sham session (ΔΔ SLEEPINESS).

#### Electrical Field Simulation

SimNIBS (Windhoff et al., 2013; Thielscher et al., 2015) was used to model the electrical field induced by tACS for each subject. First, for each subject, a head model was calculated using T1- and T2-weighted (if available) images. The head models contained five compartments (scalp, skull, gray matter, white matter and CSF) and used a mesh resolution of 0.5 nodes per mm^2^. In the second step, diffusion tensor imaging (DTI) data were integrated into the head model. Simulations were performed using the following experimental parameters in SimNIBS: 2 cm round electrodes in PO7 and PO8 positions (determined via a template cap provided by SIMNIBS), 1 mA peak-to-peak current and an estimated thickness of 0.2 cm conductivity cream between electrodes and scalp. For the anatomical components, standard conductivities were used: scalp = 0.465 S/m, skull = 0.010 S/m, CSF = 1.654 S/m, gray matter = 0.275 S/m, white matter = 0.126 S/m (Wagner et al., 2004). The results of the electrical field simulations were transformed for each subject into MNI space.

A mean electrical field strength was calculated for each subject individually by averaging all simulated electrical field values covered by a predefined mask. In the next step these averaged values were used as a predictor in a general linear regression model. Since we were aiming to find out if the EFIELD strength predicts the aftereffect better when it is determined for regions related to alpha amplitude (EFIELD_alphaBOLD_) as compared to the maximum values that reach the occipital cortex (EFIELD_strong_), we used two alternative masks per subject. To determine EFIELD_strong_ the mask included 1,000 gray matter voxels for which the highest electrical field values were estimated (MASK_strong_). Alternatively, to determine EFIELD_alphaBOLD_, the mask included 1,000 occipital gray matter voxels with the strongest negative correlation between alpha amplitude and BOLD signal (MASK_alphaBOLD_, see section “*alpha-BOLD correlation”*).

#### Analysis of Variance

To evaluate differences within and between sessions for alpha amplitude, sleepiness and DMN connectivity, we performed a two-way Analysis of Variance (ANOVA) with the two within-subject factors *session* (tACS and sham) and *run* (*pre* and *post*) for each of these measures.

#### Analysis of Covariance

To test for a difference between sessions (tACS and sham) while considering sleepiness as a covariate, we calculated an Analysis of Covariance (ANCOVA) for alpha amplitude and DMN connectivity strength. For this analysis we subtracted the *pre* recording values from the *post* recording values and calculated the ANCOVA with the factor *session* (tACS and sham) and included ΔSLEEPINESS as a covariate.

#### Multiple Linear Regression Model

To disentangle the effect of tACS on the alpha amplitude and the mPFC-PCC connectivity strength from those effects that appeared because of changes in sleepiness, multiple linear regression models were calculated. The models included the covariate ΔΔ SLEEPINESS and the two tACS-related predictors, electrical field strength (EFIELD) and frequency mismatch (MISMATCH). Frequency mismatch was calculated for each subject as the absolute difference between the tACS frequency and the mean IAF during the tACS session.


(1) ΔΔALPHA_AMP∼β×1ΔΔSLEEPINESS+β×2EFIELD+β×3MISMATCH+β4

(2) ΔΔCONNECTIVITY∼β×1ΔΔSLEEPINESS+β×2EFIELD+β×3MISMATCH+β4

#### Leave-One-Out Cross-Validation

To test the generalizability of our models, we used a cross-validation technique. Leave-one-out cross-validation (LOOCV) is a validation method that is used on small datasets like our cohort of 18 subjects. For the validation, the model is fitted *n* times (*n* = number of all observations) on *n-1* data points to predict the data point that was left out in each case. The root-mean-square error (RMSE) obtained between predicted and observed data points quantifies the generalizability of the original model to a new dataset.

**FIGURE 2:**
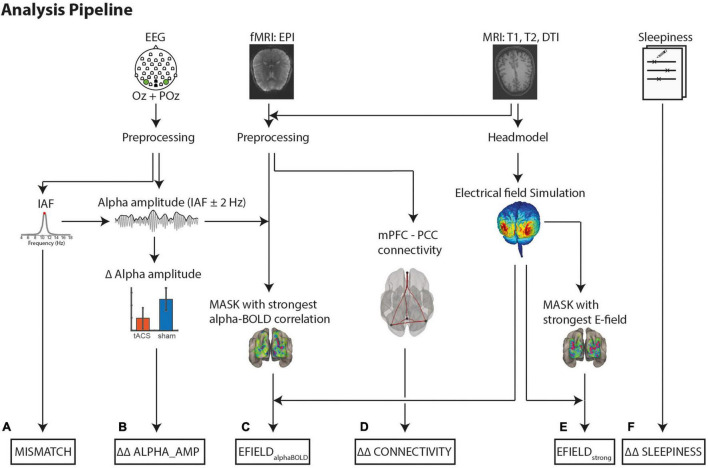
Analysis pipeline to determine the variables for a multiple linear regression model. **Dependent Variables:** **(B)** Difference (*tACS* - *sham*) in alpha amplitude change (*post* - *pre*) (ΔΔ ALPHA_AMP). Alpha amplitude was calculated as the mean envelope (absolute values of the complex Hilbert transform ± 2 Hz around IAF). **(D)** Differences (*tACS* - *sham*) in mPFC-PPC connectivity strength changes (*post* - *pre*) (ΔΔ CONNECTIVITY). **Covariate to control for intrinsic alpha modulators:** **(F)** Differences (*tACS* - *sham*) of changes (*post* - *pre*) in self-rated sleepiness (ΔΔ SLEEPINESS). **Variables to explain tACS related effects:** **(C)** EFIELD_alphaBOLD_: Mean electrical field strength was calculated individually in 1,000 occipital voxels with strongest negative alpha-BOLD correlation **(E)** EFIELD_strong_: Mean electrical field strength over 1,000 voxels with the strongest field strength. **(A)** Absolute Mismatch between tACS and IAF (|freq_tACS_ – freq_IAF_|).

Figure 2 shows an overview of the analysis steps.

## Results

All 22 subjects participated in both the tACS and sham sessions. Four subjects had to be excluded from further analysis because of a non-identifiable peak in the alpha range (8–12 Hz) during the main experiment (video watching).

### Within and Between Session Effects of the Group

In a first analysis step we investigated the effect of tACS on the alpha amplitude, mPFC-PCC connectivity strength and self-rated sleepiness by calculating a two-way ANOVA with the factors *run* (levels: *pre* and *post*) and *session* (levels: tACS and sham). ANOVA assumptions were validated by testing the residuals for homogeneity of variance (Levene’s test) and for normal distribution (Shapiro–Wilk-Test). All criteria were met within each group of both factors (*tacs* and *sham* for the factor “condition” and *pre* respectively *post* for the factor “run”). Based on previous findings, we expected to find an increase in the alpha amplitude from *pre* to *post* in both sessions (Benwell et al., 2019). In addition to this increase, alpha amplitude and the mPFC-PCC connectivity strength were expected to be positively affected by the stimulation and thus to show a stronger increase during the tACS session from *pre* to *post* compared with the sham session (Clancy et al., 2022), resulting in a significant *run × session* interaction.

**FIGURE 3:**
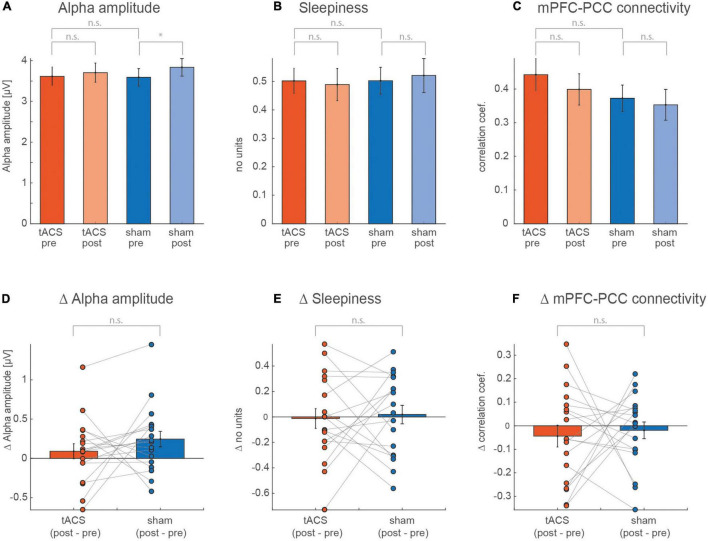
Values averaged over all subjects for *pre* and *post* of **(A)** alpha amplitude, **(B)** self-rated sleepiness, and **(C)** mPFC-PCC connectivity strength. A two-way ANOVA was calculated for each of the measurements, with the factors session (*tACS*, *sham*) and run (*pre*, *post*). **(D–F)** Show individual and averaged changes from *pre* to *post* separately for each session.

As expected, we found a significant effect of the factor *run* (*p* = 0.036) (Figures 3A,D) on the alpha amplitude. However, a *post hoc* paired t-test showed a significant increase from *pre* to *post* run during sham (*t*_17_ = –2.413, *p* = 0.03) but not during tACS (*t*_17_ = –0.921, *p* = 0.37), while we expected a stronger amplitude increase after real stimulation. The mPFC-PCC connectivity strength, illustrated in Figures 3C,F, showed a decrease from *pre* to *post* run that appears to be stronger for the tACS session. However, none of these trends reached statistical significance (see Table 1). Values for self-rated sleepiness are illustrated in Figures 3B,E and show, as expected, no significant interaction between run and session (*F*_1,16_ = 0.09, *p* = 0.77). The data also do not support an expected increase for sleepiness during the two sessions (*F*_1,16_ = 0.00, *p* = 0.96). Mean sleepiness changes for tACS session (post–pre) = –0.0128, *SD* = 0.34 and for sham session (post–pre) = 0.018, *SD* = 0.31. The *F* and *p* values of the calculated ANOVAs are listed in Table 1.

**TABLE 1:** Results of the ANOVA.

	*Run*	*Session*	*Run × Session*
*Alpha amplitude*	*F*_1,16_ = 5.16; ***p*** = 0.04*	*F*_1,16_ = 0.18; *p* = 0.68	*F*_1,16_ = 1.32; *p* = 0.27
*Sleepiness*	*F*_1,16_ = 0.00; *p* = 0.96	*F*_1,16_ = 0.10; *p* = 0.75	*F*_1,16_ = 0.09; *p* = 0.77
*mPFC-PCC connectivity*	*F*_1,16_ = 2.01; *p* = 0.17	*F*_1,16_ = 2.44; *p* = 0.14	*F*_1,16_ = 0.11; *p* = 0.74

*Significant (p < 0.05).

A tACS-induced difference in the alpha amplitude and mPFC-PCC connectivity could be masked by a concurrent brain state-induced difference (e.g., sleepiness). An ANCOVA testing the difference between conditions (tACS, sham) while considering sleepiness as a covariate, was calculated for the alpha amplitude and the mPFC-PCC connectivity strength. Neither the alpha amplitude (*F*_1,16_ = 1.08, *p* = 0.31), nor for mPFC-PCC connectivity strength (*F*_1,16_ = 0.11, *p* = 0.74) revealed a significant difference between the conditions.

### Inter-Subject Variation of the Electrical Field Strength

**FIGURE 4:**
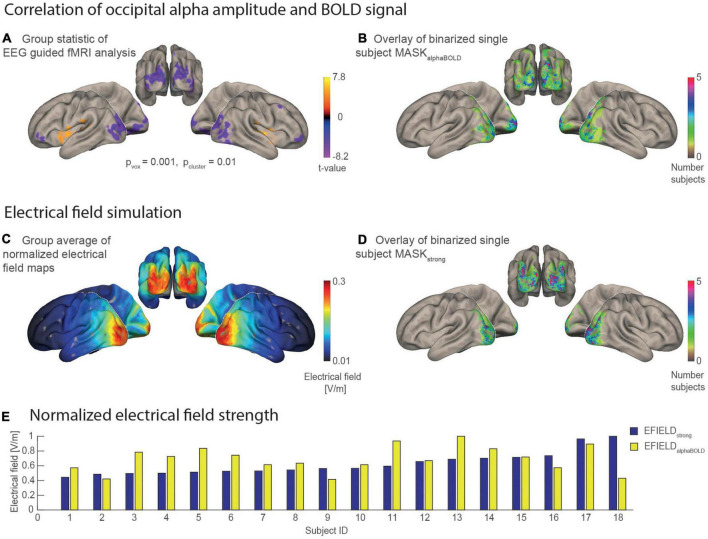
Statistical maps and masks resulting from the EEG guided fMRI analysis and the electrical field simulations mapped on the MNI brain. **(A)** Result of the group statistic calculated for the EEG-guided fMRI analysis shows the spatial distribution of positive and negative correlations between alpha amplitude and BOLD [*n* = 18, *p*(voxel level) = 0.001, *p*(cluster, fdr corrected) = 0.01]. **(B)** Overlay of 18 binary single subject masks, each covering 1,000 occipital voxels with the individually highest alpha BOLD correlation (*t*-value). The color scale codes the number of individual masks that cover the same region. **(C)** Grand average of the individual electrical field simulations (*n* = 18). **(D)** Overlay of 18 binary single subject masks that each cover a region with the 1,000 highest electrical field values. **(E)** Bar plot for the electrical field strength values of each subject that was determined as an average of simulated electrical field values covered by a subject-specific mask. EFIELD_strong_ (blue) is sorted in ascending order.

The EFIELD strength that finally reaches the brain in each subject is a crucial factor in explaining the individual stimulation effect on the alpha amplitude (Kasten et al., 2019). Using EFIELD simulations, including individual head models, we obtained a map with an electrical field value for every voxel in the brain. A field map with the field strength values averaged over all subjects is given in Figure 4C and shows, as expected, the strongest field values in the occipital-parietal region. To include a meaningful electrical field value for every subject in our model, we averaged all field values covered by a predefined mask (MASK_strong_ and MASK_alphaBOLD_). MASKs_alphaBOLD_ were individually defined by 1,000 occipital gray matter voxels for which the alpha amplitude shows the strongest negative correlation with the BOLD signal. To achieve the best signal-to-noise ratio in calculating these masks, the first level results of the EEG-guided fMRI analysis were pooled over all runs and sessions for each subject. This assumes no spatial difference of the alpha-BOLD correlation between the two sessions or runs. A two-way ANOVA indeed showed no significant effects for *session, run* nor a significant interaction between *session x run* on a level of *p*_*voxel*_ = 0.005, *p*_*cluster*_ = 0.05 (FDR-corrected for multiple comparisons). Figure 4A shows the map of the second level group statistic, with the strongest negative correlation covering the left and right occipital-parietal regions.

Figures 4B,D, respectively, each show an overlay of the single subject masks on the inflated surface of the MNI brain. The subject-wise MASKs_alphaBOLD_ are spatially distributed more heterogeneously between subjects compared with the subject-wise MASKs_strong_. This means that brain areas covered by MASKs_alphaBOLD_ vary more strongly between subjects (thus are more subject-specific), while the MASKs_strong_ cover rather similar brain areas for the subjects. The bar plot in Figure 4E illustrates for each subject the electrical field strength that was averaged in the area of MASK_strong_ (EFIELD_strong_) compared with MASK_alphaBOLD_ (EFIELD_alphaBOLD_). Values of EFIELD_strong_ are, on average, higher (mean = 0.27, *SD* = 0.07) than EFIELD_alphaBOLD_ (mean = 0.12, *SD* = 0.03), while, for both measures, the standard deviation was about ± 25%. The inter-subject variance of the mean electrical field strength differed between the two masks. For example, the subject with the highest value for EFIELD_strong_ did not necessarily have the highest value in alpha related-areas (EFIELD_alphaBOLD_) compared with the other participants.

### Mismatch of Transcranial Alternating Current Stimulation- and Individual Alpha Frequency

Previous studies suggest that stimulation frequency and brain oscillation frequency should match for efficient entrainment of the intrinsic oscillations (Stecher et al., 2017; Stecher and Herrmann, 2018; Kasten et al., 2019). The stimulation frequency was, therefore, adjusted for all subjects to their dominant frequency peak in the occipital alpha range (8–12 Hz), which had been determined in the eyes-closed recording done before the main experiment. However, the dominant alpha peak during video watching (main experiment) and eyes-closed was not always the same within a subject (Supplementary Figure S1B). Furthermore, the highest power peak in the alpha range could slightly shift from *pre* to *post* during video watching (Supplementary Figure S1C), as is also expected from the literature (Benwell et al., 2019). Thus, many subjects received stimulation with a frequency slightly above or below their current IAF during the stimulation run. The histogram in Supplementary Figure S2A shows the divergence of the applied tACS frequency from IAF (averaged for *pre* and *post* of the tACS session) for all subjects during video watching. On average, IAF and tACS show a mismatch of 0.3 Hz (median) while 83% of the subjects had a mismatch of less than 0.8 Hz. The largest mismatch was 1.3 Hz.

### Multiple Linear Regression Model

Our aim was to explain the inter-subject variability of tACS aftereffects on the alpha amplitude and the mPFC-PCC connectivity strength in a linear regression model. ‘We considered individual factors, which are related to the tACS application, and also at individual covariates to account for variances due to changes in subject’s mental states. In the end, our models considered, as tACS related predictors, the individual electrical field strength, the frequency mismatch, and the change of self-rated sleepiness as a covariate. We expected that the individual change in sleepiness explains a significant amount of inter-subject variance in the alpha modulation (from *pre* to *post*) (Zhao et al., 2012; Wascher et al., 2014), as well as changes in mPFC-PCC connectivity strength (from *pre* to *post*) (Pomares et al., 2019). In addition, we expected a significant contribution of electrical field strength and frequency mismatch, where subjects with a higher electrical field in the gray matter masks were expected to have a stronger aftereffect on both alpha amplitude and mPFC-PCC connectivity strength (Kasten et al., 2019; Johnson et al., 2020). Furthermore, we expected a reduction in aftereffect (alpha amplitude as well as mPFC-PCC connectivity strength) for a higher mismatch between IAF and applied tACS frequency (Stecher and Herrmann, 2018; Kasten et al., 2019).

**FIGURE 5:**
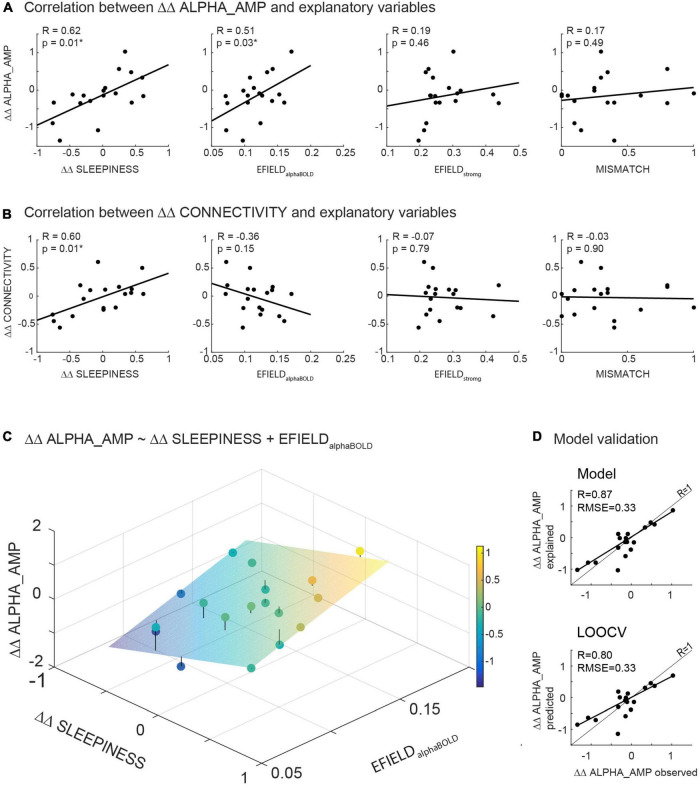
**(A,B)** Illustrates the results of correlations calculated between the readout variables (ΔΔ ALPHA_AMP resp. ΔΔ CONNECTIVITY) and potential modulating variables (ΔΔ SLEEPINESS, MISMATCH, EFIELD_alphaBOLD_ and EFIELD_strong_). **(C)** Illustrates the result of the significant model (ΔΔ ALPHA_AMP ∼ΔΔ SLEEPINESS + EFIELD_alphaBOLD_) in a three-dimensional plot: The scatter plot represents the observed ΔΔ ALPHA_AMP, while the plane shows the calculated ΔΔ ALPHA_AMP based on the parameter estimated in the model. **(D)** Depicts the observed ΔΔ ALPHA_AMP in relation to estimated ΔΔ ALPHA_AMP based on the model fit (upper part) resp. to ΔΔ ALPHA_AMP predicted by the leave-one-out cross validation (LOOCV) (below).

Since we determined the EFIELD field strength in two alternative ways (EFIELD_alphaBOLD_ and EFIELD_strong_), we fitted two multiple linear regression models to each of the dependent variables (ΔΔ ALPHA_AMP resp. ΔΔ CONNECTIVITY). The results of the four model fits are listed in Table 2. In all four models, ΔΔ SLEEPINESS contributed significantly (*p* < 0.05) to explaining the variation of the dependent variable (ΔΔ ALPHA_AMP resp. ΔΔ CONNECTIVITY), while MISMATCH had no explanatory contribution in any of the models (*p* > 0.05). This behavior of the two variables is in line with the results when they were separately correlated with the dependent variables (Figures 5A,B). The two variables representing alternative values for the individual electrical field strength (EFIELD_strong_ and EFIELD_alphaBOLD_) contributed differently to the models. EFIELD_alphaBOLD_ contributed highly significantly (*p* < 0.05) in the model that explained the variance of ΔΔ ALPHA_AMP, but not in the model that considered ΔΔ CONNECTIVITY as dependent variable. EFIELD_strong_, however, had no significant effect on any of the dependent variables (*p* > 0.05). The particular contribution of EFIELD_stron*g*_ respectively EFIELD_alphaBOLD_ in the models is in line with the respective correlations in Figures 5A,B. The only significant correlation was found between EFIELD_alphaBOLD_ and ΔΔ ALPHA_AMP. The results of the models show that none of the tACS-related factors (EFIELD, MISMATCH) were significantly involved in explaining the ΔΔ CONNECTIVITY. In contrast, we found a highly significant relationship between EFIELD_alphaBOLD_ and ΔΔ ALPHA_AMP. Removing MISMATCH from the model, since it did not explain any variance, the full model equation reads as follows:


(3) ΔΔALPHA_AMP=0.9×ΔΔSLEEPINESS+11.7×EFIELD-alphaBOLD1.5

**TABLE 2:** Results of the multiple linear regression models.

Model equation	*p*-Values	Model *p*-Value	Model *R*^2^	Model RMSE	LOOCV RMSE
ΔΔ ALPHA_AMP ∼		0.0002**	0.70	0.31	0.37
ΔΔ SLEEPINESS +	0.0001**				
MISMATCH +	0.66				
EFIELD_alphaBOLD_	0.0005**				

ΔΔ ALPHA_AMP ∼		0.025*	0.36	0.46	0.52
ΔΔ SLEEPINESS +	0.005**				
MISMATCH +	0.76				
EFIELD_strong_	0.18				

ΔΔ CONNECTIVITY ∼		0.036*	0.33	0.25	0.30
ΔΔ SLEEPINESS +	0.013*				
MISMATCH +	0.68				
EFIELD_alphaBOLD_	0.19				

ΔΔ CONNECTIVITY ∼		0.078	0.24	0.27	0.33
ΔΔ SLEEPINESS +	0.012**				
MISMATCH +	0.62				
EFIELD_strong_	0.84				

*Significant (p < 0.05), **Significant after correction for multiple comparisons (p < 0.0125).

Figure 5C illustrates the results of the model (eq. 3) in a three-dimensional plot. The plane in the coordinate system depicts the estimated ΔΔ ALPHA_AMP for any pair of ΔΔ SLEEPINESS and EFIELD_alphaBOLD_ based on the model fit. The mean distance of all recorded data points from the plane, resulting in the *Root Mean squared error* (RMSE), reflects the quality of the model fit. A combination of ΔΔ SLEEPINESS and EFIELD_alphaBOLD_ (without MISMATCH) explains 72% (adjusted *R*^2^ = 0.72) of the inter-subject variance of ΔΔ ALPHA_AMP with an RMSE of 0.31.

To independently quantify the contribution of sleepiness and EFIELD to the alpha aftereffect, we first regressed out sleepiness before correlating EFIELD and alpha amplitude change. As expected, the correlation between EFIELD_alphaBOLD_ and ΔΔ ALPHA_AMP is stronger after regressing out ΔΔ SLEEPINESS (*R* = 0.51 before, *R* = 0.76 after). Same for EFIELD_strong_ and ΔΔ ALPHA_AMP (*R* = 0.19 before, *R* = 0.36 after).

The results did not change significantly, when we calculated the model separately for tACS and sham sessions (Δ ALPHA_AMP ∼Δ SLEEPINESS + EFIELD_alphaBOLD_). For the tACS session the model explained 46% of the variance in Δ ALPHA_AMP, while EFIELD_alphaBOLD_ contributed significantly (*p* = 0.006). However, for the sham session only 7% of the data were explained with no significant contribution of EFIELD_alphaBOLD_ (*p* = 0.49). Based on these results, we conclude that the EFIELD is really the most significant factor that explains the amplitude modulation during tACS session.

### Validation of the Model

To evaluate our model (eq. 3) with regard to generalizability (predictability in a new dataset) and exclude the possibility of overfitting, we calculated a leave-one-out cross-validation. Subsequently, the observed data (ΔΔ ALPHA_AMP) were correlated with the data predicted by LOOCV (Figure 5D bottom), as well as the data estimated by the model (Figure 5D top). Comparing these correlations, the error between predicted and observed data is not greater than the error between estimated and observed data (both RMSE = 0.31). As expected, the model fits the current dataset slightly better than it predicts data points with LOOCV. Altogether, the validation method confirmed the quality of our model (Figure 5D).

## Discussion

The current study investigated the aftereffects of alpha-tACS applied over the occipito-parietal cortex on alpha EEG amplitude, as well as on mPFC-PCC connectivity strength as measured by fMRI. Contrary to our expectations, an analysis of variance revealed no significant tACS effect on the alpha amplitude or on mPFC-PCC connectivity strength. Further, an analysis of covariance that considers sleepiness as a concurrent intrinsic factor that could influence alpha amplitude and probably also mPFC-PCC connectivity strength, besides the possible effect of tACS, also did not reveal a difference between tACS and sham sessions. Considering a parameter for subject-specific EFIELD strength in addition to subjective sleepiness in a multiple linear regression model, we were able to verify a significant increasing aftereffect of tACS. The effect was found only on the alpha amplitude, and not on the mPFC-PCC connectivity strength.

### Group Mean: Analysis of Variance

The ANOVA did not reveal a significant tACS effect on the alpha amplitude, which differs from the findings of several previous studies that reported increased occipital alpha amplitude after tACS (Zaehle et al., 2010; Neuling et al., 2013; Helfrich et al., 2014; Vossen et al., 2015; Kasten et al., 2016). Zaehle et al. (2010), for instance, applied alpha-tACS on the occipital cortex using an electrode montage similar to ours (PO9/PO10). Even though they had a smaller number of subjects (ten per group), they found a significantly greater increase in the alpha power of the stimulation group compared to the sham group. However, several other studies were also not able to detect an aftereffect of tACS on the alpha amplitude (Veniero et al., 2017; Stecher and Herrmann, 2018). Stecher and Herrmann (2018), among others, applied tACS with durations of 1, 3, 4, and 10 min. After each block, they measured the resting-state EEG for 10 min during a visual vigilance task. They were unable to find a lasting enhancement of alpha power following any stimulation block when the tACS groups were compared with the sham group. Nevertheless, when they examined the mismatch between stimulation frequency and IAF they found a significant effect of stimulation following 10 min of α-tACS.

Furthermore, in our present study, an ANOVA calculated on the connectivity strength between mPFC-PCC showed no significant differences for a change from *pre* to *post* between sham and tACS sessions. In contrast, a recent study with 41 subjects reported a significant increase in connectivity strength between the same two medial DMN nodes (mPFC and PCC) after tACS application (Clancy et al., 2022). However, several factors, including stimulation duration, stimulation strength and the electrode montages, were different compared to our study. Clancy et al. (2022), for example, stimulated considerably longer than we did (20 min instead of 7 min). In our previous fMRI study (Cabral-Calderin et al., 2016) we also stimulated the occipital cortex for a shorter period, i.e., 8 min, using a P5/P6 montage, and did not find a significant tACS effect on the DMN connectivity strength, which is similar to the findings in our current dataset. Therefore, the tACS effect of a shorter stimulation period (7 resp. 8 min) could be, in fact, just too weak to induce a measurable change in connectivity strength between mPFC and PCC.

### Consideration of Individualized Parameters to Model Inter-Subject Variances in Transcranial Alternating Current Stimulation Aftereffects

Inter-individual differences are frequently mentioned and discussed as further explanations for the contradictory results between the various tACS studies (Neuling et al., 2013; Tavakoli and Yun, 2017; Cabral-Calderin and Wilke, 2019). The differences in individual head anatomies, for example, can lead to different EFIELD distributions and strengths in the target regions (Antonenko et al., 2021). In addition, subjects show differences in alpha frequency stability during a given experiment, which results in more or less mismatch between the stimulation frequency and the intrinsic alpha frequency. The frequency stability itself is highly dependent on the intrinsic stability of the subject’s brain states and also on the ongoing task demands during tACS application (Benwell et al., 2019). Therefore, better explanatory models that take individual factors into consideration and neither under- nor overestimate the effect of tACS are needed.

In a recent magnetoencephalography (MEG) study, Kasten et al. (2019) presented a multiple linear regression model that explained a large amount (76%) of the inter-subject variability in the observed alpha power increase after tACS application over the visual cortex. Their model incorporated the individual EFIELD strength, the individual mismatch between IAF and tACS frequencies, and the spatial overlap of the EFIELD distribution and the target region (estimated alpha generators). In contrast to our data, this study detected a significant tACS aftereffect on the alpha amplitude using permutation cluster *t*-tests on source level alpha power. Since multiple tACS parameters of our study differed from their study (e.g., electrode montage, stimulation duration, task), several reasons could explain this discrepancy. One possibility is that the effect induced by tACS was much weaker in our study, due to the shorter stimulation duration (7 min instead of 20 min), and thus the effect might have been covered by the natural amplitude changes occurring spontaneously during the experiment. Based on this hypothesis, we expanded the model introduced by Kasten et al. (2019) with an additional factor (sleepiness) to account for natural amplitude changes in the alpha frequency band. In the end, we established a multiple linear regression model consisting of a combination of individual EFIELD strength, frequency mismatch and changes of self-rated sleepiness.

In our study, we found a positive correlation of the individual ΔΔ ALPHA_AMP with both the individually determined EFIELD_alphaBOLD_ and the self-rated ΔΔ SLEEPINESS, suggesting an influence of both factors on the intrinsic alpha amplitude. This was confirmed by the highly significant results of a multiple linear regression model, which explained 70% of the ΔΔ ALPHA_AMP variance between subjects, using EFIELD_alphaBOLD_, frequency MISMATCH and ΔΔ SLEEPINESS as predictors. Since EFIELD_alphaBOLD_ had a highly significant contribution in our model, this result supports previous findings reported by Kasten et al. (2019) and emphasizes that the individual EFIELD strength has a strong influence on stimulation-induced alpha amplitude changes. This finding also supports the importance of considering individual stimulation parameters to detect a tACS aftereffect on the alpha amplitude, which might have remained undetected otherwise.

Apart from alpha amplitudes, changes in mPFC-PCC connectivity strength (ΔΔ CONNECTIVITY) also correlated positively with changes in self-rated sleepiness (ΔΔ SLEEPINESS). Applying the same multiple linear regression model (using EFIELD_alphaBOLD_, MISMATCH and ΔΔ SLEEPINESS as predictors) yielded a significant contribution only for ΔΔ SLEEPINESS to explain the inter-subject variance of ΔΔ CONNECTIVITY. None of the tACS-related factors, EFIELD and MISMATCH, contributed significantly to the variance. We were thus unable to detect any changes related to stimulation on the connectivity strength between mPFC and PCC, as shown by Clancy et al. (2022), neither with conventional group statistics (ANOVA) or with advanced statistical methods. Possible reasons could be that the tACS effects on connectivity are smaller than those on the EEG amplitude.

### Influence of Individualized Parameters in Explaining the Variance of the Transcranial Alternating Current Stimulation Aftereffect

The results of our model showed that the increase in alpha amplitude after tACS application is the result of a linear combination of the individual EFIELD strength in the target regions (EFIELD_alphaBOLD_) and changes in sleepiness (ΔΔ SLEEPINESS).

#### Electrical Field Strength

Although all subjects were stimulated with 1 mA on the scalp, the strength of the EFIELD that finally reached the brain varied strongly between subjects due to the individual anatomy and resulting conductivity (Thielscher et al., 2015; Antonenko et al., 2021). This consideration makes the individual EFIELD strength a crucial factor in explaining inter-subject variability in the tACS-related amplitude increase. The result of our model shows that the individual EFIELD strength is significantly positively related to an increase in alpha amplitude from *pre* to *post*. This relation was even stronger when sleepiness as an intrinsic alpha amplitude modulating factor was regressed out before EFIELD and alpha amplitude were correlated. This is in line with previous findings, which showed that a stronger EFIELD induces a stronger tACS aftereffect (Johnson et al., 2020).

However, it needs to be established how to utilize the mean EFIELD strength based on the individual simulation maps. For example, we averaged the values of the simulated EFIELD strength in two alternative regions of the occipital cortex: MASK_alphaBOLD_ and MASK_strong_. The accuracy of our model improved greatly when the individual EFIELD strength was determined in brain areas that are related to alpha activity (EFIELD_alphaBOLD_), compared with averaging the strongest EFIELD values for each subject (EFIELD_strong_). Consistent with these results, Kasten et al. (2019) showed in their model the importance of considering the overlap of regions with the strongest induced EFIELD and regions estimated to be alpha generators. In order to identify alpha generating areas, Kasten performed a source estimation based on high density MEG data, while we determined alpha-related occipital areas via an alpha amplitude-guided fMRI analysis. Although they used different analyses approaches, both studies show the importance of considering the EFIELD strength in brain areas that are related to the alpha amplitude. Thus, for describing the efficiency of tACS on the alpha amplitude, it might be beneficial to not only determine the maximum EFIELD values for each subject, but rather include the values of the EFIELD strength that exist in the target region, in our case alpha-related areas.

To calculate the mean EFIELD from the simulated maps, Kasten et al. (2019) incorporated an area consisting of 10,000 voxels, including gray and white matter voxels, while we decided to incorporate a much smaller area (1,000 voxels encompassing only gray matter), because our approach was planned to study alpha-specific areas. However, recalculating our models using EFIELD strengths averaged over 10,000 gray matter voxels led to similar results (see Supplementary Table S1). Since both area sizes (1,000 and 10,000 voxels) are somewhat arbitrary, the optimal size for the EFIELD strength calculations remains an open issue.

#### Sleepiness as a Concurrent Factor

Transcranial alternating current stimulation-related aftereffects on the alpha amplitude might be undetected or misinterpreted in cases where the natural alpha modulation arising from continuous changes in brain state, e.g., due to increased sleepiness, covers the tACS-induced amplitude change. To disentangle alpha amplitude changes due to mental state changes from those induced by tACS, we assessed self-rated sleepiness as an independent variable that represents an alpha-related brain state. Our results imply that including self-rated sleepiness in a linear regression model can reveal tACS-related amplitude effects that would have otherwise remained undetected. Nevertheless, calculating an ANCOVA and testing the difference between conditions (tACS, sham) while considering sleepiness as a covariate did not reveal significant differences between conditions on the alpha amplitude and also not on the mPFC-PCC connectivity strength. These results support the importance of additionally considering the EFIELD for proving the effectiveness of tACS as discussed above. Although EFIELD showed a significant correlation with ΔΔ ALPHA_AMP, even this correlation was much stronger after regressing out the alpha amplitude effects based on changes in sleepiness. Thus, sleepiness could be a crucial factor in observing a tACS effect targeting the alpha amplitude in experiments, which without stimulation presumably have a strong change of the alpha amplitude based on mental brain changes (e.g., exhausting task or dark environment). It is also possible that sleepiness is particularly important in studies in which subjects are in a supine position, e.g., in the MRI scanner.

#### Frequency Mismatch

The absolute mismatch between the stimulation frequency and the IAF (mean IAF during the tACS session) had no significant contribution in explaining the variability of tACS-induced aftereffects in our model, neither for alpha amplitude changes nor for mPFC-PCC connectivity changes. This means that, in our study, the offset between stimulation frequency and IAF had no influence on the tACS aftereffect.

Previously, only a few tACS studies took the possible influence of frequency mismatch on the tACS aftereffect into account, with contradictory results (Vossen et al., 2015; Stecher et al., 2017; Stecher and Herrmann, 2018; Kasten et al., 2019). Stecher, for instance, reported a relationship between frequency mismatch and aftereffect on the alpha amplitude in their study (Stecher and Herrmann, 2018). Kasten showed a significant contribution of frequency mismatch in a model explaining inter-subject variance in tACS-induced alpha amplitude increase (Kasten et al., 2019). They considered negative and positive values for the frequency mismatch (IAF above or below stimulation frequency), and they found a stronger tACS aftereffect with stimulation frequencies slightly above IAF. Vossen et al. (2015), on the contrary, suggested that a stimulation frequency below the IAF had a stronger aftereffect on the alpha amplitude.

From a theoretical perspective, a perfect match between the tACS frequency and IAF should indeed matter: A common hypothesis about the tACS mechanism is that intrinsic oscillations are entrained during tACS application, leading to an increase in alpha amplitude. This entrainment requires less tACS intensity if the frequencies are close to each other (Pikovsky et al., 2001). However, it should be noted that in most of the studies, including ours, the mismatch was calculated between the stimulation frequency and a mean of IAF determined before and after stimulation (EEG recording is difficult during stimulation due to the large artifacts induced by tACS). Thus, we cannot really know the true offset and difference between IAF and stimulation frequency during tACS application. In this context, it is also necessary to mention that the subject’s IAF is generally not stable during any experimental procedure (see also Supplementary Figure S1) because the dominant peak frequency varies depending on the mental state. A perfect match between the IAF and tACS at coinciding time points would require a continuous adjustment of the stimulation frequency in a closed loop system. However, even when an intermittent closed loop stimulation protocol is employed, the results might not be optimal. In a recent study, the parietal alpha rhythm was targeted using a closed loop system. The hypothesis was that the closer match of intrinsic and stimulation frequency should lead to an increased detection of visual luminance changes depending on a stronger effect on the alpha peak power in comparison with a protocol using a fixed stimulation frequency (Stecher et al., 2021). The results showed that only the fixed stimulation protocol led to a persistent increase in post-stimulation alpha power compared with sham. Our results also suggest that since the effectiveness of the stimulation did not depend on a perfect match between intrinsic alpha frequency and tACS frequency, such a technical effort might be unnecessary. It should also be noted that in the previously mentioned studies the frequency mismatch was larger than in the present study, which might explain why this factor did not contribute to the variance in our model. Furthermore, Kasten et al. (2019) also considered interactions between frequency mismatch and the strength of the EFIELD. From a conceptual perspective, it makes sense that the frequency mismatch can only have an effect on the alpha amplitude if the EFIELD is strong enough and vice versa. This interaction was not considered in the present study.

In summary, the results of the present study support the importance of individually calculated EFIELD strength along with self-rated sleepiness as two crucial factors that can explain and predict a large amount of inter-subject variability of the alpha amplitude change after tACS applied over occipito-parietal cortex. We found that determining the EFIELD strength in individual, alpha-related brain areas has an advantage over just averaging the individual maximum current strength values. In future studies, individualized dose-control could probably eliminate the variance in EFIELD intensities at a cortical target site. Supposing that the current delivered to the brain directly determines its behavioral consequences, this method may allow for reducing the known variability of tACS effects.

## Data Availability Statement

The original contributions presented in this study are included in the article/Supplementary Material, further inquiries can be directed to the corresponding author/s.

## Ethics Statement

The studies involving human participants were reviewed and approved by University Medical Center Göttingen, Germany. The patients/participants provided their written informed consent to participate in this study.

## Author Contributions

IS and KW developed the study conception and collected the data. IS analyzed and interpreted the data. IS, MW, and AA discussed the results. IS and AA drafted the manuscript. KW and MW proofread the manuscript and provided critical comments. All the authors approved the final version of the manuscript.

## Conflict of Interest

The authors declare that the research was conducted in the absence of any commercial or financial relationships that could be construed as a potential conflict of interest.

## Publisher’s Note

All claims expressed in this article are solely those of the authors and do not necessarily represent those of their affiliated organizations, or those of the publisher, the editors and the reviewers. Any product that may be evaluated in this article, or claim that may be made by its manufacturer, is not guaranteed or endorsed by the publisher.
