# Supplementary material for: Detection of Transcranial Alternating Current Stimulation Aftereffects Is Improved by Considering the Individual Electric Field Strength and Self-Rated Sleepiness
Source: Front Neurosci. 2022 Jun 27;16:870758. doi: 10.3389/fnins.2022.870758 (PMC9272587; doi:10.3389/fnins.2022.870758)
Supplement: Supplementary file 1 [file Data_Sheet_1.docx]

Supplementary Material

# Supplementary Data, Figures and Tables


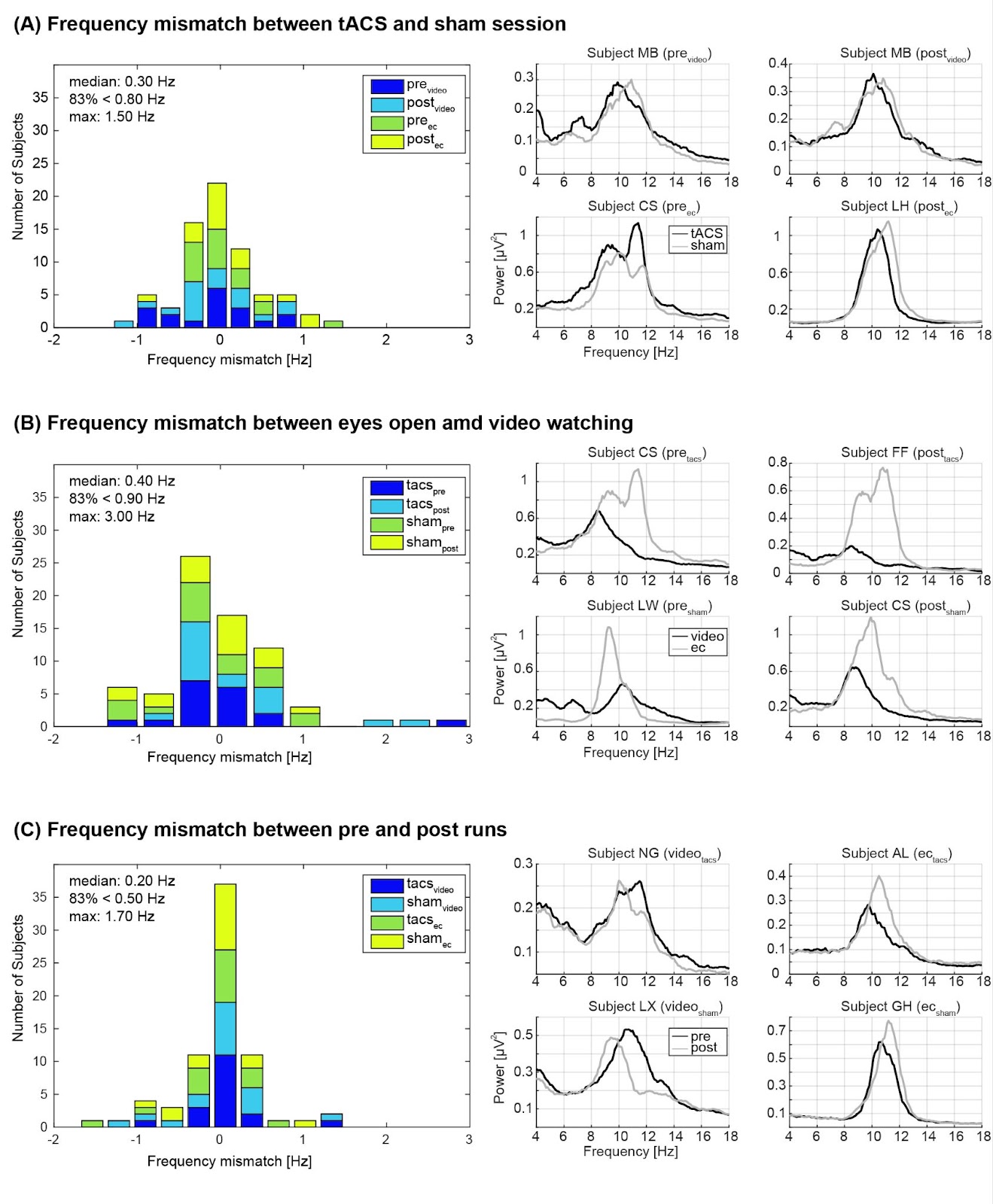


**Figure S1:** Mismatch between IAFs that were determined under different experimental conditions: **(A)** IAF mismatch between tACS and sham session, **(B)** IAF mismatch between pre and post runs, **(C)** IAF mismatch between eyes-closed (ec) and video watching (video). Histograms on the left side of the figure illustrate the distribution of IAF mismatches over the subjects, while colors refer to the composition of the plotted data. The right side illustrates the frequency mismatch between the highest peaks in the frequency spectra for some example subjects.


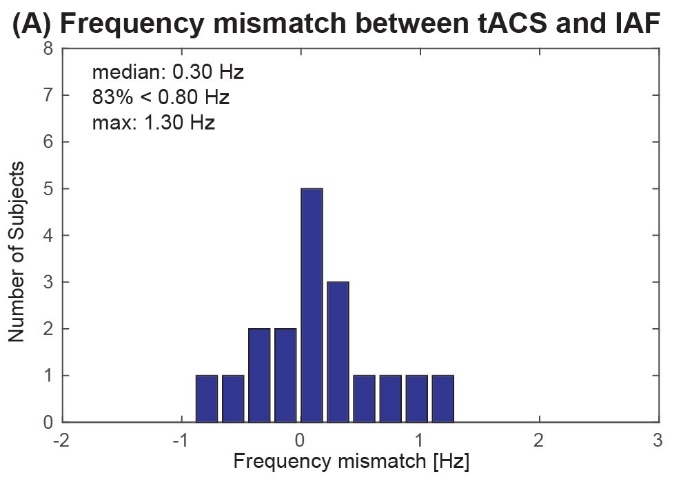


**Figure S2:** Histogram of frequency mismatch between stimulation frequency and the mean IAF of the tACS session ( (IAF_pre_ + IAF_post_ ) / 2)

*Table S1: Results of the multiple linear regression models (electrical field strength averaged over 10,000 voxel)*

| **Model**  **equation** | **p-Values** | **Model**  **p-Value** | **Model**  **R^2^** | **Model**  **RMSE** | **LOOCV**  **RMSE** |
| --- | --- | --- | --- | --- | --- |
| ∆∆ ALPHA_AMP ~  ∆∆ SLEEPINESS +  MISMATCH +  EFIELD_alphaBOLD_ | **0.0002****  0.23  **0.001**** | **0.0003**** | 0.67 | 0.33 | 0.39 |
| ∆∆ ALPHA_AMP ~  ∆∆ SLEEPINESS +  MISMATCH +  EFIELD_strong_ | **0.001****  0.19  **0.008** | **0.002*** | 0.56 | 0.38 | 0.42 |
| ∆∆ CONNECTIVITY ~  ∆∆ SLEEPINESS +  MISMATCH +  EFIELD_alphaBOLD_ | **0.013***  0.56  0.43 | 0.06 | 0.27 | 0.26 | 0.32 |
| ∆∆ CONNECTIVITY ~  ∆∆ SLEEPINESS +  MISMATCH +  EFIELD_strong_ | **0.012***  0.62  0.87 | 0.078 | 0.24 | 0.27 | 0.31 |

**Significant ( p < 0.05), **Significant after correction for multiple comparisons (p < 0.0125)*
